# Upregulation of long non-coding RNA *LOC284454* may serve as a new serum diagnostic biomarker for head and neck cancers

**DOI:** 10.1186/s12885-020-07408-w

**Published:** 2020-09-24

**Authors:** Chunmei Fan, Jinpeng Wang, Yanyan Tang, Shanshan Zhang, Fang Xiong, Can Guo, Yanhong Zhou, Zheng Li, Xiaoling Li, Yong Li, Guiyuan Li, Zhaoyang Zeng, Wei Xiong

**Affiliations:** 1 Department of Stomatology, NHC Key Laboratory of Carcinogenesis, Xiangya Hospital, Central South University, No.88 Xiangya Road, Changsha, Hunan P. R. China 410078; 2 The Key Laboratory of Carcinogenesis and Cancer Invasion of the Chinese Ministry of Education, Cancer Research Institute and School of Basic Medicine, Central South University, Changsha, Hunan China; 3 Hunan Key Laboratory of Nonresolving Inflammation and Cancer, Disease Genome Research Center, The Third Xiangya Hospital, Central South University, Changsha, Hunan China; 4 Department of Medicine, Dan L Duncan Comprehensive Cancer Center, Baylor College of Medicine, Houston, TX USA

**Keywords:** Head and neck cancers, Long noncoding RNAs, Receiver operating characteristic, Serum biomarker

## Abstract

**Background:**

Identification of effective diagnostic and prognostic biomarkers of cancer is necessary for improving precision medicine. Long non-coding RNAs (lncRNAs) play an important regulatory role in tumor initiation and progression. The lncRNA *LOC284454* is distinctly expressed in various head and neck cancers (HNCs), as demonstrated by our previous bioinformatics analysis. However, the expression levels and functions of *LOC284454* in cancer are still unclear.

**Methods:**

We investigated the dysregulation of lncRNAs in HNCs using the GEO database and found that *LOC284454* was highly expressed in HNCs. Serum samples from 212 patients with HNCs and 121 normal controls were included in this biomarker study. We measured the expression of *LOC284454* in the sera of HNC patients and normal controls using RT-qPCR. Receiver operating characteristics (ROC) analysis is an important statistical method that is widely used in clinical diagnosis and disease screening. ROC was used to analyze the clinical value of *LOC284454* in the early diagnosis of HNCs.

**Results:**

*LOC284454* was significantly upregulated in the sera of patients with nasopharyngeal carcinoma, oral cancer, and thyroid cancer. *LOC284454* upregulation had good clinical diagnostic value in these cancers, as evaluated by area under the ROC curve values of 0.931, 0.698, and 0.834, respectively.

**Conclusions:**

*LOC284454* may be a valuable serum biomarker for HNCs facilitating the early diagnosis of malignant cancers. Further studies are needed to elucidate the mechanisms underlying the involvement of *LOC284454* in HNCs. This study provides the first evidence that *LOC284454* may be a serum biomarker for HNCs.

## Highlights

1. This is the first study examining *LOC284454* expression in serum of HNCs patients.
2. This study provides the first evidence of *LOC284454* as a serum biomarker for HNCs.

## Background

Head and neck cancers (HNCs), including cancers of the oral cavity, tongue, hypopharynx, nasopharynx, larynx, and thyroid, are the sixth most common cancers worldwide, with an estimated incidence of more than 500,000 new cases each year [15, 51, 52]. Most patients are in an advanced stage of HNCs at the time of diagnosis, with cervical lymph node involvement and/or distant metastasis. In these patients, the risk of metastasis and recurrence is significantly increased, and the mortality rate rises sharply.

Effective biomarkers for early diagnosis and prognosis are important for reducing the mortality of HNCs. Liquid biopsy is currently an effective and non-invasive method. Some serum markers, such as Epstein Barr virus DNA and microRNAs (miRNAs), lactate dehydrogenase, and antigens have been recognized for their clinical value [8, 10, 36, 38, 39, 42, 43, 48]. However, they also have some limitations. Identifying serum biomarkers with high sensitivity and specificity is an urgent goal.

Long non-coding RNAs (lncRNAs) are transcripts longer than 200 nucleotides that most of them do not encode proteins [4, 17–19, 21, 53, 58]. In recent years, many studies have shown that a variety of lncRNAs are frequently expressed in malignant cancers and may participate in the initiation and development of malignant cancers [9, 28, 30–32, 45, 49, 57]. For example, the AFAP1-AS1 lncRNA promotes the proliferation, migration, and invasion of cervical cancer, colon cancer and nasopharyngeal carcinoma (NPC) through different mechanisms [2, 3, 29]. Additionally, *PVT1* lncRNA induces radioresistance by regulating DNA repair and cell apoptosis, while promoting the proliferation of thyroid cancer through polycomb enhancer of zeste homolog 2/thyroid-stimulating hormone receptor [23, 25, 47]. However, the functional importance of most lncRNAs has not yet been elucidated, including their roles in human tumors. Only a few lncRNAs have been reported to have clinical implications for early screening and prognosis.

Presently, we examined the expression level of *LOC284454* in patients’ serum with HNCs and evaluated its clinical significance as a serum biomarker for early diagnosis.

## Methods

### Sample collection

We used blood collection tubes containing anticoagulants, mixed gently after blood collection. The samples were centrifuged at 1000–3000 rpm for 10 min, the supernatant was collected for RNA extraction. Blood samples were transported on ice and stored in − 80 °C refrigerator. Hemolysis and hyperlipidemia samples during blood collecting and low quality RNA during RNA extraction were excluded. Unbiased both men and women patients were included, who had not received any radio-chemotherapy or surgery before diagnosis. In total, 333 serum samples were collected from Affiliated Cancer Hospital of Central South University within 2017. This study was approved by the Ethical Committee of Central South University. Written informed consent was obtained from all patients and healthy donors.

### Patients’ enrollment

The samples were collected from 121 normal donors randomly and 212 HNC patients. Of the 212 HNC serum, 100 were NPC, 55 were oral cancer, and 57 were thyroid cancer serum samples. Sex and age distribution were summarized in Supplemental table 1.

### RNA extraction and real-time quantitative polymerase chain reaction (RT-qPCR)

Serum RNA was extracted using miRNeasy Serum/Plasma Kit (Qiagen, Germany). Since our commen use housekeping genes may change its expression in tumor serum, thus we introduced an external reference, pGL3 [5]. The pGL3 (1 ng, approximately 2 × 10^8^ copies) was added to serum samples according to the manufacturer’s protocol using an miRNeasy Serum/Plasma Kit (Qiagen, Germany). The extracted serum RNA was reverse transcribed using a Revert Aid First Strand cDNA Synthesis Kit (Thermo Fisher Scientific, USA). Forward (F) and reverse (R) primers were synthesized by TSINGKE Biological Technology Company (China), as follows: *LOC284454*-F, 5′-ATTACAGGTGGCTCAGGTGT-3′, *LOC284454*-R, 5′-CTTCAGTGTGCCTCCTCAGT-3′; and pGL3-F, 5′-TCCATCTTGCTCCAACACCC-3′, pGL3-R, 5′-TCGTCTTTCCGTGCTCCAAA-3′. The probe sequences were as follows: *LOC284454*-P, 5′-FAM-CGTGCCTGGCTTTTCTCCACTATCTTG-BHQ1–3′ and pGL3-P, 5′-HEX-ACGCAGGTGTCGCAGGTCTTCC-BHQ1–3′. Conventional SYBR-qPCR was performed using iTaq universal SYBR Green Supermix (Bio-Rad, USA). TaqMan-qPCR was performed using iTaq Universal Probes Supermix (Bio-Rad,USA). All RT-qPCR procedures were performed using a Bio-Rad CFX96 Multicolor Real-time PCR Detection System. TaqMan-qPCR allowed the simultaneous detection of two probes in the same tube (Bio-Rad, USA).

### Statistical analysis

GSE61218 is from our group, which aims to identify significantly expressed lncRNAs in NPC tissues. GSE68799 is a RNA-Seq data identified human transcriptome alterations in NPC. RNA-Seq has been proved a tool with high throughput and coverage, reliable accuracy. GSE53819 is a genome-wide expressing profiling of NPC included 18 NPC tissue samples versus 18 control samples. They are paired tumor tissues and non-cancerous controls, which we thought can reduce individual heterogeneity. After discovering that *LOC284454* is highly expressed in NPC, we also wanted to know whether it is highly expressed in other head and neck cancers, so we randomly selected the GEO dataset of oral cancer (GSE30784) and thyroid cancer (GSE33630). Data were analyzed using SPSS 13.0 (SPSS Inc., USA) and GraphPad Prism 7.0 (GraphPad, USA). Student’s t-tests were used to evaluate differences between two groups of samples. Normal distribution was analysed via Graphpad Prism 7, D’Agootino-Pearson, Kolmogoov-Smirnov, or Shapiro-Wilk were used to test whether the data conforms to the normal distribution, if *p* > 0.1, we can use Student t-test, if no, we may use Non-parametric Wilcoxon test. *P*-values< 0.05 were considered statistically significant. Correlation with clinic-pathological variables were evaluated through spearman or pearson correlation test. All the results obtained were from three independent replicates. The area under the curve (AUC), sensitivity, and specificity were obtained by receiver operating characteristic (ROC) curve analysis.

## Results

### *LOC284454* is upregulated in NPC, oral cancer, and thyroid cancer

**Fig. 1:**
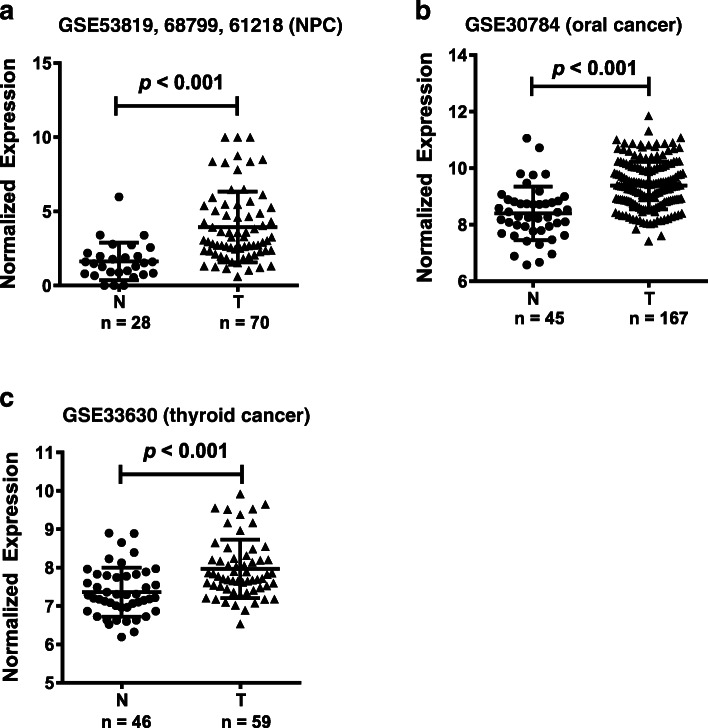
Upregulation of *LOC284454* in head and neck cancers in the GEO database. *LOC284454* was significantly upregulated in several head and neck cancers, including nasopharyngeal carcinoma (**a**), oral cancer (**b**), and thyroid cancer (**c**)

We explored the dysregulation of lncRNAs in HNCs using the GEO database. *LOC284454* was significantly upregulated in several cancers, including NPC, oral cancer, and thyroid cancer. In our previous article, we performed gene expression profiling enrolled six inflammatory normal controls and 10 NPC tissues to identify differentially expressed lncRNAs (accession number GSE61218). Forty-six thousand five hundred six lncRNA probes were included. The data showed that totally 1276 lncRNAs were differentially expressed, including 405 upregulated and 871 downregulated lncRNAs in NPC tissues. We selected top 20 highly expressed lncRNAs to validate. *LOC284454* was one of the most significant and had not been detected its application as a serum biomarker (heatmap showed in Fig. 1a) [11]. In NPC, we integrated three sets of gene expression profiles, including GSE53819, GSE68799, GSE61218, which further demonstrated that *LOC284454* is highly expressed in NPC. GSE30784 and GSE33630 were used to analyze oral cancer and thyroid cancer, respectively. The expression levels of *LOC284454* were significantly higher in NPC (Fig. 1a, *P* < 0.001), oral cancer (Fig. 1b, *P* < 0.001), and thyroid cancer (Fig. 1c, *P* < 0.001), compared to non-tumor tissues.

### *LOC284454* expression is significantly increased in serum of patients with NPC

**Fig. 2:**
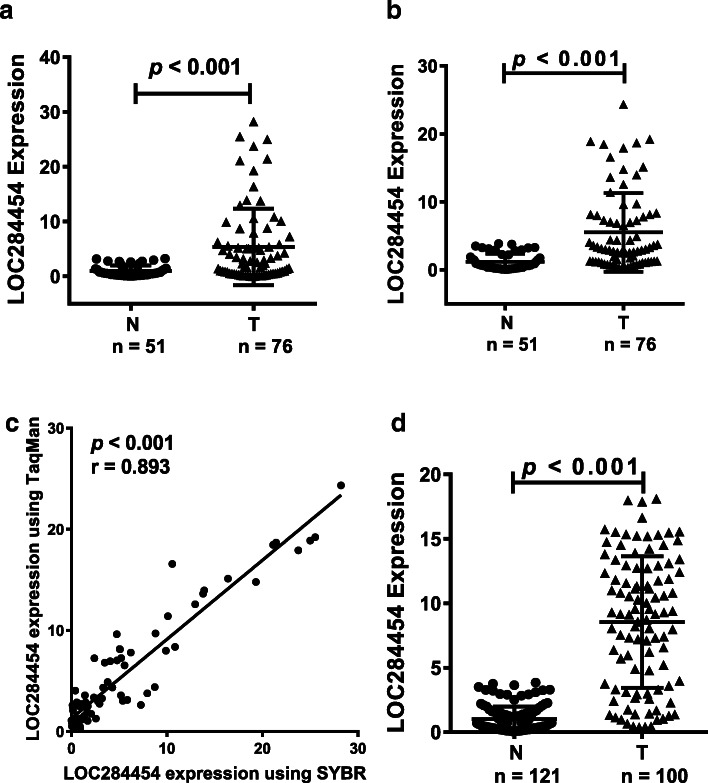
*LOC284454* expression is significantly higher in serum of patients with NPC. **a**. SYBR Green qPCR assay was used to detect the expression of *LOC284454* in the serum of 76 NPC patients and 51 healthy donors. **b**. A TaqMan probe for *LOC284454* was used to detect the expression of *LOC284454* in the same samples. **c**. Correlation analysis of the results obtained by SYBR-qPCR and TaqMan-qPCR. **d**. Verification of the expression of *LOC284454* in 100 NPC and 121 normal control samples

SYBR-qPCR was used to detect the expression of *LOC284454* in the serum of 76 NPC patients and 51 healthy donors. *LOC284454* expression level was significantly higher in the serum of patients with NPC (Fig. 2a, *P* < 0.001).

Next, to eliminate systematic errors and make the results more reliable, we designed a TaqMan probe for *LOC284454* and tested the same serum samples using TaqMan-qPCR. This examination also revealed significantly higher expression of *LOC284454* in the serum of the NPC patients (Fig. 2b, *P* < 0.001). This was consistent with previous conventional RT-qPCR results. Subsequent correlation analysis of the results obtained by SYBR-qPCR and TaqMan-qPCR demonstrated a good positive correlation between the two methods, which verified the reliability of this data (Fig. 2c, *P* < 0.001).

We also verified the expression of *LOC284454* in a larger cohort of 121 normal controls and 100 NPC patients (added some new samples to the original cohort). The expression of *LOC284454* in the serum of NPC patients was significantly higher than that of the normal control group (Fig. 2d, *P* < 0.001). Taken together, these results suggested that *LOC284454* may be a potential serum marker for NPC.

### *LOC284454* is highly expressed in serum of patients with oral cancer and thyroid cancer

**Fig. 3:**
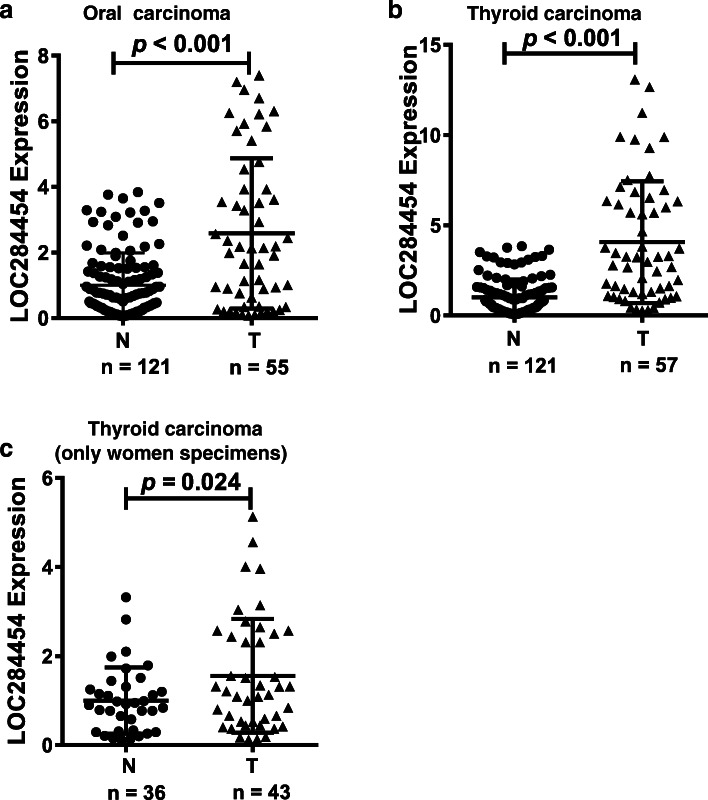
*LOC284454* is highly expressed in serum of patients with oral cancer and thyroid cancer. Using TaqMan-qPCR to detect the expression of *LOC284454* in oral cancer (**a**) and thyroid cancer (**b**). (**c**). Expression of *LOC284454* in 43 female thyroid cancer patients and 36 normal women

The results of the GEO database results suggested that *LOC284454* may be dysregulated in oral cancer and thyroid cancer. Therefore, we next detected the expression of *LOC284454* in the serum of patients with these cancers using TaqMan-qPCR. *LOC284454* was significantly upregulated in the serum of patients with oral cancer and thyroid cancer compared with those of the normal controls (Fig. 3a & b, *P* < 0.001). Notably, a significant difference was evident in the proportion of men and women with thyroid cancer (43 females and 14 males). To exclude gender effects, we analyzed the expression of *LOC284454* in 43 female thyroid cancer patients and 36 normal women. The expression of *LOC284454* was higher in the tumor serum than in the normal group (Fig. 3c, *P* = 0.024). The collective results demonstrated that the expression level of *LOC284454* in the serum of patients with oral and thyroid cancers was significantly higher than normal controls.

### Diagnostic value of serum *LOC284454* for HNC patients

ROC is commonly used to assess the diagnostic value of biomarkers. AUC refers to the area enclosed by the curve and the 45-degree diagonal line, which is used to quantify the diagnostic value. An AUC value < 0.5 indicates almost no diagnostic value. AUCs of 0.5 ~ 0.7, 0.7 ~ 0.9, and > 0.9 indicate low, moderate, and high diagnostic value, respectively. Values exceeding 0.9 indicate high specificity and sensitivity. When the sensitivity and specificity are the largest, we select this point as the best cut-off point. Diagnostic values of *LOC284454* in these three kind of head and neck cancers are shown in Table 1. To improve the the quality of reporting diagnostic accuracy in this study, we followed the STARD statement.

**Table 1:** Diagnostic values of LOC284454 in head and neck cancers

Cancer	NPC	Oral cancer	Thyroid cancer
Cut-off	3.65	3.29	3.29
Sensitivity	74.00	65.00	69.00
Specificity	97.52	95.87	95.87
PPV	96.10	92.86	98.57
NPV	81.94	76.82	78.91
FDR	3.90	7.14	1.43
FNR	18.06	23.78	21.09
Accuracy	86.88	81.90	83.71
LH+	29.84	15.74	16.71
LH-	0.27	0.37	0.32

*PPV* positive predictive values, *NPV* negtive preditive value, *FPR* false positive rate, *FNR* false negtive rate, *LH+* positive likehood ratio, *LH-* negative likehood ratio

**Fig. 4:**
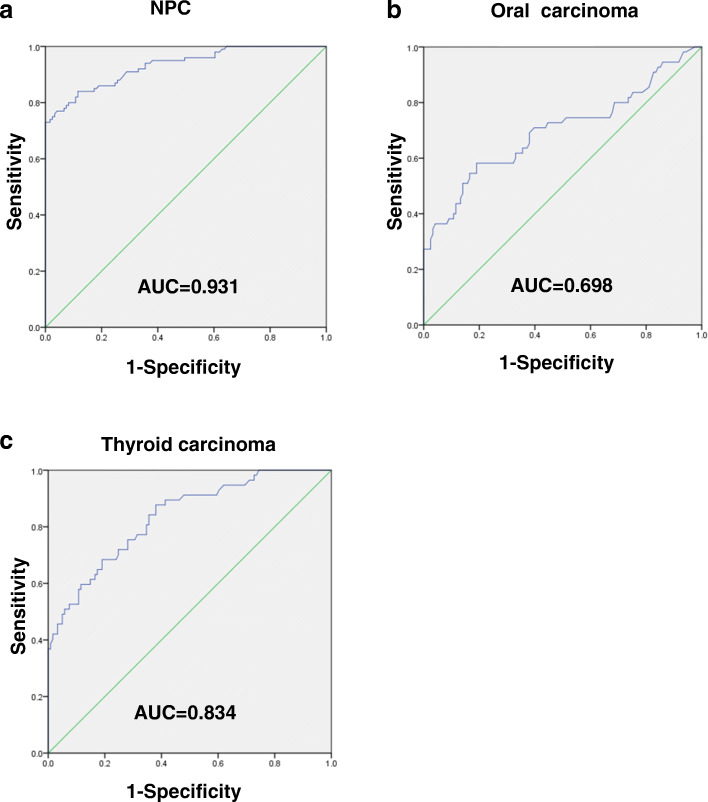
Diagnostic value of serum *LOC284454* for HNC patients ROC analysis was performed to evaluate the diagnostic value of LOC284454. The AUC values of LOC284454 in NPC (**a**), oral cancer (**b**), and thyroid cancer (**c**) were 0.931, 0.698, and 0.834, respectively

The AUC values of *LOC284454* in NPC (Fig. 4a), oral cancer (Fig. 4b), and thyroid cancer (Fig. 4c) were 0.931, 0.698, and 0.834, respectively, indicating that *LOC284454* might be an appropriate diagnostic biomarkers for these cancers (Table 2). However, we analyzed the *LOC284454* expression level as well as patients’ clinical characteristics and found that no correlation was observed between *LOC284454* and pathological stages, or gender, or age distribution.

**Table 2:** ROC curves analysis of LOC284454 in nasopharyngeal carcinoma, oral cancer and thyroid cancer

	Area	Std. Error^a^	Asymptotic Sig.^b^	Asymptotic 95% Confidence Interval			
cancer types				Lower Bound	Upper Bound	Sensitivity	Specificity
nasopharyngeal carcinoma	0.931	0.017	0.000	0.899	0.964	0.740	0.975
oral cancer	0.698	0.047	0.000	0.606	0.791	0.650	0.959
thyroid cancer	0.834	0.032	0.000	0.771	0.898	0.690	0.959

^a^Under the nonparametric assumption

^b^Null hypothesis: true area = 0.5

## Discussion

HNCs rank as the sixth most common type of cancers worldwide. The cancers are often at an advanced stage at the time of diagnosis and display frequent recurrence and metastasis. Thus, prognosis and patient survival are poor. Radiotherapy and chemotherapy have largely improved the treatment of HNCs in recent decades [12, 33, 34, 41, 50, 56]. However, the 5-year survival rate is still very low. Improving the accuracy of early diagnosis could significantly improve the disease-free survival rate of patients.

Compared with other detection methods, liquid biopsy has become the preferred choice for disease screening because of its non-invasiveness, low cost, ease of use, and high stability. Some biomarkers for HNCs, including proteins, miRNAs, and EBV DNA, have been identified using liquid biopsies [13, 54]. However, each of these markers has its own disadvantages, including low positive rates, high false positive rate, need for experienced operators, and instrumental limitations. Therefore, finding effective early diagnostic markers in serum is critical for the treatment of HNCs.

LncRNAs have been reported to participate in the pathogenesis of HNCs. LncRNAs circulating in the serum or other bodily fluids present promising biomarkers for clinical diagnostic and prognostic applications. For example, serum *MALAT1*, *AFAP1-AS1*, and *AL359062* can function as diagnostic and prognostic biomarkers for NPC [22]. Notably, the upregulation of the *ATB* lncRNA can accurately predict papillary thyroid carcinoma and its prognosis [6]. However, few studies have examined novel lncRNAs expression in serum in HNCs.

The *LOC284454* lncRNA is located on 19p13.12 and the miR-23-a ~ 27a ~ 24–2 cluster is present upstream of the same transcript. *LOC284454* is a nuclear localized and chromatin associated lncRNA. *LOC284454* RNA is found only in primates and is highly conserved. In our previous study, we demonstrated that *LOC284454* promotes migration and invasion of NPC cells in vitro and in vivo, and is associated with skeletal remodeling and adhesion signal pathways [11]. In this study, based on the feasibility of SYBR-qPCR and TaqMan-qPCR tests of serum *LOC284454*, we found that compared with healthy controls, the expression of *LOC284454* was higher in NPC, oral cancer, and thyroid cancer, indicating that *LOC284454* might be very important for the diagnosis of HNCs. To confirm this, we used ROC curve analysis to evaluate the diagnostic value of *LOC284454*. The AUC values of *LOC284454* in NPC, oral cancer, and thyroid cancer were 0.931, 0.698, and 0.834, respectively, indicating that *LOC284454* might be an appropriate diagnostic biomarker for these cancers. Even though we found that *LOC284454* is highly expressed in NPC, oral cancer, and thyroid cancer, that does not mean *LOC284454* can be generalized to all cancers. Study have shown that *LOC284454* is significantly reduced in prostate, uterus, breast, and kidney cancer [7], suggesting that *LOC284454* is specificly highly expressed in HNC.

Real-time PCR can sensitively detect small changes in nucleic acids based on fluorescent dyes and fluorescently labeled probes. In TaqMan-PCR, a fluorescent reporter group and a fluorescence quenching group are labeled on both ends of the probe [1, 14, 16, 55]. When amplified, the 5′-3′ exonuclease activity of the Taq enzyme degrades the probe. The fluorescent reporter group and the fluorescence quenching group are separated, so that the fluorescence monitoring system can receive the fluorescent signal, and the accumulation of fluorescent signal is completely synchronized with the formation of the PCR product [20, 24, 37]. Since the qPCR instrument has a multicolor fluorescent channel, the experimental group and the control group are allowed to react in the same tube with the same cDNA template, which can reduce systematic errors and improve the specificity and sensitivity of the experiment [40]. This is also one of the highlights of this study and might be very useful for future detection of biomarkers.

We found that *LOC284454* is highly expressed in the peripheral blood of HNCs. Why it remains stable in the peripheral blood is still unclear. We suspect that this may be related to exosomes or vesicles. Exosomes can encapsulate proteins, lipids, and nucleic acids, remain stable in the tumor microenvironment, and are important in tumor metastasis [46]. Recent studies have shown that non-coding RNAs exist in exosomes. Exosomes can carry non-coding RNAs to non-adjacent cells for information communication and participate in tumor development [26, 27, 35, 44]. More research is needed to elucidate these mechanisms.

In summary, our results verified that *LOC284454* is significantly upregulated in the serum of patients with NPC, oral cancer, and thyroid cancer based on SYBR-qPCR and TaqMan-qPCR. Moreover, ROC curve data indicates that *LOC284454* could be used as a novel diagnostic biomarker for HNCs. Further research should focus on follow-up investigations to study the prognostic value of *LOC284454*. It is hoped that the development of new technologies, such as digital PCR, will make it easier to detect phenotypic specific molecular changes, and will increase the sensitivity and specificity of biomarkers.

## Conlusions

In this study, we investigated the dysregulation of lncRNAs in HNCs using the GEO database and found that *LOC284454* was highly expressed in HNCs (nasopharyngeal carcinoma, oral cancer, and thyroid cancer). We measured the expression of *LOC284454* in the serum of HNC patients via Taqman RT-qPCR. We then used ROC curve to analyze the clinical value of *LOC284454* in the early diagnosis of HNCs. *LOC284454* upregulation had good clinical diagnostic value in nasopharyngeal carcinoma, oral cancer, and thyroid cancer, as evaluated by area under the ROC curve values of 0.931, 0.698, and 0.834, respectively. *LOC284454* may be a valuable serum biomarker for HNCs facilitating the early diagnosis of malignant cancers. Further studies are needed to elucidate the mechanisms underlying the involvement of *LOC284454* in HNCs. This study provides the first evidence that *LOC284454* may be a serum dipgnostic biomarker for HNCs.

## Supplementary information

**Additional file 1: Table S1.** Statistical analysis of the sex and age distribution of head and neck cancer and normal control group.

## Data Availability

The expresion data of *LOC24454* was aquired from GEO datasets (https://www.ncbi.nlm.nih.gov/), the accession numbers are GSE61218, GSE68799, GSE53819, GSE30784, GSE33630.

## Abbreviations

LncRNAs: Long non-coding RNAs
HNCs: Head and neck cancers
ROC: Receiver operating characteristics
miRNA: MicroRNA
NPC: Nasopharyngeal carcinoma
AUC: The area under the curve
