# Supplementary material for: Upregulation of long non-coding RNA LOC284454 may serve as a new serum diagnostic biomarker for head and neck cancers
Source: BMC Cancer. 2020 Sep 24;20:917. doi: 10.1186/s12885-020-07408-w (PMC7517628; doi:10.1186/s12885-020-07408-w)
Supplement: Supplementary file 1 — Additional file 1: Table S1. Statistical analysis of the sex and age distribution of head and neck cancer and normal control group. [file 12885_2020_7408_MOESM1_ESM.docx]

**Supplemental table 1. Statistical analysis of the sex and age distribution of**

**head and neck cancer and normal control group**

| Group | gender | number /（ percent） | *P^a^*value | average age | *P^b^* value |
| --- | --- | --- | --- | --- | --- |
| normal control | male | 85（70.2％） |  | 42.4±13.0 |  |
|  | female | 36（29.8％） |  | 40.6±11.4 |  |
|  | total | 121（100％） |  | 41.8±12.5 |  |
| nasopharyngeal carcinoma | male | 77（77％） |  | 48.1±8.7 | 0.140 |
|  | female | 23（23％） |  | 41.6±12.5 | 0.741 |
|  | total | 100（100％） | 0.259 | 46.6±10.0 | 0.499 |
| oral  carcinoma | male | 46（83.6％） |  | 52.3±10.6 | 0.490 |
|  | female | 9（16.4％） |  | 46.7±15.3 | 0.224 |
|  | total | 55（100％） | 0.059 | 51.6±11.2 | 0.055 |
| thyroid cancer | male | 14（24.6％） |  | 43.4±11.6 | 0.054 |
|  | female | 43（75.4％） |  | 43.7±12.7 | 0.252 |
|  | total | 57（100％） | ＜0.001 | 43.6±12.3 | 0.055 |

Note: *P^a^* represents the difference between the sex ratio of male and female in each tumor group and the normal control group.

*P^b^* represents the age difference between male patients, female patients or general patients in each tumor group and the corresponding comparison of the normal control group.
